# Developing a Predictive Tool for Hospital Discharge Disposition of Patients Poststroke with 30-Day Readmission Validation

**DOI:** 10.1155/2021/5546766

**Published:** 2021-08-19

**Authors:** Jin Cho, Krystal Place, Rebecca Salstrand, Monireh Rahmat, Misagh Mansouri, Nancy Fell, Mina Sartipi

**Affiliations:** ^1^Department of Computer Science and Engineering, University of Tennessee at Chattanooga, USA; ^2^Center for Urban Informatics and Progress, University of Tennessee at Chattanooga, USA; ^3^Department of Physical Therapy, University of Tennessee at Chattanooga, USA

## Abstract

After short-term, acute-care hospitalization for stroke, patients may be discharged home or other facilities for continued medical or rehabilitative management. The site of postacute care affects overall mortality and functional outcomes. Determining discharge disposition is a complex decision by the healthcare team. Early prediction of discharge destination can optimize poststroke care and improve outcomes. Previous attempts to predict discharge disposition outcome after stroke have limited clinical validations. In this study, readmission status was used as a measure of the clinical significance and effectiveness of a discharge disposition prediction. Low readmission rates indicate proper and thorough care with appropriate discharge disposition. We used Medicare beneficiary data taken from a subset of base claims in the years of 2014 and 2015 in our analyses. A predictive tool was created to determine discharge disposition based on risk scores derived from the coefficients of multivariable logistic regression related to an adjusted odds ratio. The top five risk scores were admission from a skilled nursing facility, acute heart attack, intracerebral hemorrhage, admission from “other” source, and an age of 75 or older. Validation of the predictive tool was accomplished using the readmission rates. A 75% probability for facility discharge corresponded with a risk score of greater than 9. The prediction was then compared to actual discharge disposition. Each cohort was further analyzed to determine how many readmissions occurred in each group. Of the actual home discharges, 95.7% were predicted to be there. However, only 47.8% of predictions for home discharge were actually discharged home. Predicted discharge to facility had 15.9% match to the actual facility discharge. The scenario of actual discharge home and predicted discharge to facility showed that 186 patients were readmitted. Following the algorithm in this scenario would have recommended continued medical management of these patients, potentially preventing these readmissions.

## 1. Introduction

Determining discharge disposition after stroke is a complex decision-making process by the healthcare team. After index hospitalization in a short-term acute care hospital, patients may be discharged to their home or another facility for continued medical or rehabilitative management. Many factors affect a patient's discharge destination, including patient-related factors such as age, race, comorbidities, and functional status [1] as well as healthcare system-related factors such as bed availability and workforce [2]. In the acute care hospital, the healthcare team works together with the patient and the patient's family to determine whether the patient can return home or requires transfer to another facility. The site of postacute care has effects on overall mortality [3] and 6-month functional outcomes in the domains of basic mobility, activities of daily living, and applied cognition [4]. The process of determining discharge destination is often delayed by insurance approval, rehabilitation assessment, and medical management, thus increasing the patient's length of stay, risk of infection, and unnecessary costs. Early prediction of discharge destination may optimize poststroke care and improve outcomes by mitigating these delays. While many have attempted to predict discharge disposition after stroke [1, 5–7], outcomes are limited to validate whether the prediction was truly appropriate for the patient in a clinically meaningful way.

Hospital readmission is one metric of quality of care and discharge planning. Low readmission rates indicate the proper and thorough care with appropriate discharge disposition. Readmissions are costly to the healthcare system, averaging $14,400 per readmission and affecting 13.9% of all index hospitalizations [8]. In Medicare beneficiaries, 30-day readmission rates approached 20% at an estimated cost of $17.4 billion in 2004 [9]. With the Centers for Medicare and Medicaid Services (CMS) Hospital Readmissions Reduction Program (HRRP), hospitals receive reduced payment for services rendered for excess readmissions [10]. As this program expands [11], hospitals continue to strive to identify and address preventable readmissions. Predictive analytics is one strategy being used with other conditions to mitigate excess readmissions and reduce cost by identifying and intervening for patients who are at a high risk of readmission [12–14]. After a stroke, patients are at high risk for complications such as recurrent stroke, fractures, deep vein thrombosis, and urinary tract infections [15]. 30-day readmission rates for stroke range from 8.7% to 17.4% [16–18]. Preventing these readmissions is one of the primary goals of discharge planning in the acute care hospital. In this study, readmission status was used as a measure of the clinical significance and effectiveness of a discharge disposition prediction tool.

The purpose of this research is to create and validate a predictive tool for discharge disposition poststroke in Medicare beneficiaries from 2014 to 2015 claims. Most strokes occur in people over age 65 [19]; therefore, CMS data is well-suited for studying this patient population. The predictive tool is aimed at developing a risk score for each patient based on demographics related to stroke risk and clinical characteristics at the point of the index hospitalization. We hypothesized that patients with a higher risk score would have a higher chance of being discharged to a healthcare facility. Validation of the predictive tool was based on readmission rates when the prediction differed from the patient's actual discharge location. We hypothesized that there would be higher readmission rates when a patient was discharged home but the prediction tool recommended discharge to a facility for continued management.

## 2. Materials and Methods

### 2.1. Data

We used data from the Centers for Medicare and Medicaid Services (CMS). Our dataset includes all of the records of hospitalized patients with the principal diagnosis of stroke (International Classification of Diseases, Ninth Revision codes 430, 431, 432,0, 432.1, 432.9, 433.01, 433.11, 433.21, 433.31, 433.81, 433.91, 434.01, 434.11, 434.91, 435, 435.0, 435.1, 435.3, 436, 437.1, 437.5, 997.02). This data contains information such as patient demographics, diagnosis codes, procedure codes, and other clinical information.

#### 2.1.1. Data Cleaning

The original dataset for our study included 1,385,364 records of patient claims that were associated with beneficiary IDs that had been admitted to hospital for at least one case of primary diagnosis of stroke during January 2014 to December 2015, which were the only available records when the data was acquired. We excluded 1,275,445 records of claims with primary diagnosis other than stroke. Out of the remaining 109,919 records of claims hospitalized with a primary diagnosis of stroke, additional 35,494 records were removed (not admitted to a short-term acute care hospital: 22,777, deceased/expired during hospitalization: 6,519, and patients discharged to other locations: 6,198). Of the remaining 74,425 records, 31,625 (42.5%) corresponded to home discharge and 42,800 (57.5%) corresponded to facility discharge (Table 1).

#### 2.1.2. Feature Choices

We grouped age into three categories: 18 to 64 years, 65 to 74 years, and 75 years and older. Stroke types were pooled into three different categories: ischemic, meningeal hemorrhage, and intracerebral hemorrhage. We included diabetes, high cholesterol, obesity, hypertension, atrial fibrillation, other atrial diseases, chronic kidney disease, heart disease, peripheral arterial disease, other vascular diseases, prior stroke or TIA, acute heart attack, sleep habits, alcohol habits, drug habits, smoking, family history, depression, and other diagnoses as comorbidities or other possible risk factors. Sources of admission were grouped into five different categories: nonhealthcare facility (physician's referral), clinic referral, transfer from a hospital, transfer from a skilled nursing facility (SNF), and other facilities. Primary health insurance was divided into Medicaid or Medicare, private insurance, or other insurances. Hospital discharge disposition status was coded as home discharge when patients were discharged to home with or without home health (HH) care services and defined as facility discharge when patients were discharged to healthcare facilities such as an SNF, an inpatient rehabilitation facility (IRF), and another short-term general hospital for inpatient care.

### 2.2. Methods

#### 2.2.1. Statistical Analysis

By dividing the age into three different age groups, all the features become categorical. Pearson's Chi-square test was used to determine the independency of the features. Based on the result of the Chi-square test, no associations were found between the discharge status and different groups within each feature, considering a significant level of 0.05 (Table 1). A general collinearity test was performed to the total cohort, and no strong collinearity was observed between the different features. Based on the statistical analysis, a multivariable logistic regression model was developed; odds ratios and unadjusted odds ratios as well as their corresponding 95% confidence intervals and coefficients (betas) with a significant level of 0.05 were generated to examine the discharge status in the training cohort. Based on the values of the coefficients, different risk factors were evaluated and coded for further analysis.

### 2.3. Part A: Obtaining Risk Scores through Logistic Regression

Based on cohorts selected above, logistic regression was performed to estimate odds ratios (ORs) of patient characteristics associated with facility discharge. Both unadjusted and adjusted ORs with 95% confidence intervals were considered. After that, coefficients (beta) from the multivariate logistic regression model were utilized to derive risk scores [5, 20, 21]. A total risk score was calculated for each patient by taking the sum of corresponding risk scores (see example in Table 2). After the logistic function, the predicted probability of facility discharge for each total risk score was presented and compared against the observed counterpart. Lastly, a predictive tool was made by using the total risk score to predict the hospital discharge disposition status of each patient with a primary diagnosis of stroke.

### 2.4. Part B: 30-Day Readmission Analysis

**Figure 1:**
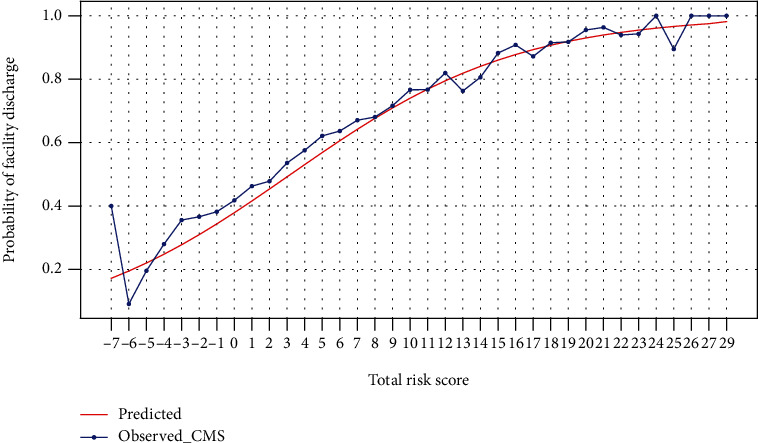
Predicted and observed probabilities of facility discharge for each total risk score.

After the total risk score was calculated for each patient, the total risk score was converted into a predicted discharged disposition status ($\hat{y}$), to be compared with the actual discharge disposition status ($\hat{y}$) for the readmission analysis. Based on the probability of facility discharge for a given total risk score (Figure 1), we established a threshold value to assign the value “home discharge” for total risk scores that are lower than the threshold value, and “facility discharge” for the scores that are greater than or equal to the threshold value (Table 3).

After the conversion step, we separated patients by their discharge disposition status (home or facility), and from there, we further broke down the data into four cases: (1) actual discharge status is home and predicted discharge status is home, (2) actual discharge status is home and predicted discharge status is facility, (3) actual discharge status is facility and predicted discharge status is home, and (4) actual discharge status is facility and predicted discharge status is facility. All four cases were tested to see if the patients returned to hospital within 30 days. A 30-day search window was applied for January 2014 to eliminate claims that were from before 2014. Furthermore, we removed any claims that were recorded after December 1st, 2015, to select cohorts strictly from 2014 to 2015. After removing data through a searching window, the dataset for our investigation included 66,172 stroke patients with unique beneficiary IDs.

All of the statistical analyses were performed using Python version 3.6 (Python Software Foundation, Wilmington, DE).

## 3. Results and Discussion

### 3.1. Results on Analysis Part A

Based on both unadjusted and adjusted ORs, patient characteristics such as female sex; ages 75 years and older; black race; meningeal hemorrhage or intracerebral hemorrhage; presence of diabetes, hypertension, atrial fibrillation, chronic kidney disease, heart disease, acute heart attack, alcohol habit, depression, or other diagnoses; and transfer from a hospital, transfer from an SNF, or other were associated with an increased risk of having a facility discharge (Table 4).

The range of the calculated risk scores for patient characteristics was from -3 to 13 (Table 5). The range of the calculated total risk score for a given patient was from -7 to 29. The predicted probability of facility discharge increased with the increase in total risk score (Figure 1), which indicates that a patient with a higher total risk score had a higher chance of being discharged to a healthcare facility.

### 3.2. Results on Analysis Part B

**Figure 2:**
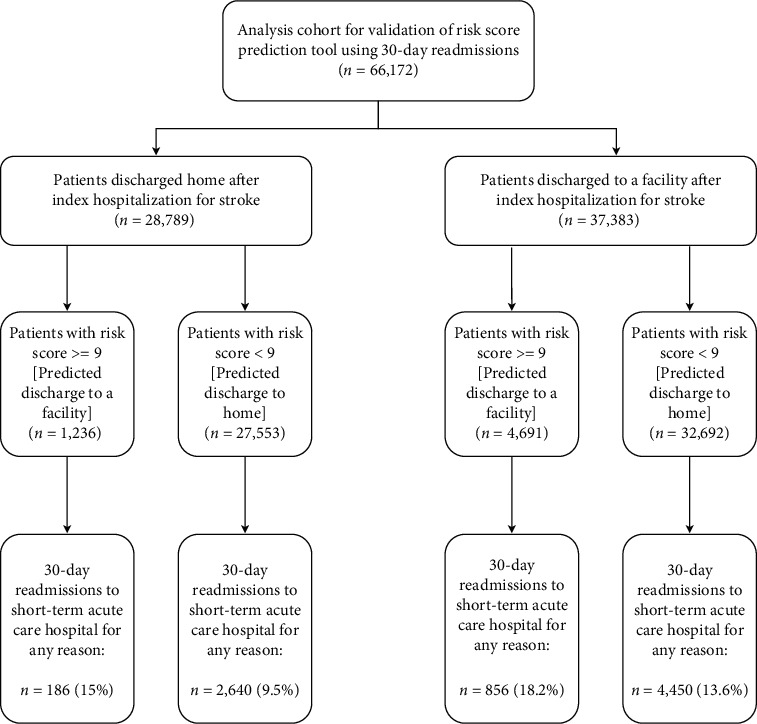
Flowchart of analysis cohort for validation of risk score prediction tool using 30-day readmission.

Out of 66,172 unique stroke patients who were being tested for 30-day readmission analysis, 28,789 (43.5%) patients were related to home discharge, and the other 37,383 (46.5%) patients corresponded to facility discharge. For the case where the actual discharge status is a home and predicted discharge is a facility (*n* = 1,236), 186 (15%) patients were readmitted within 30 days. For the case where both the actual and predicted discharge status are home (*n* = 27,553), 2,640 (9.5%) patients were readmitted within 30 days. For the case where actual discharge status is facility and predicted discharge status is facility (*n* = 4,691), 856 (18.2%) patients were readmitted within 30 days. Lastly, for the case where actual discharge status is a facility and predicted discharge is home (*n* = 32,692), 4,450 (13.6%) patients were readmitted within 30 days (Figure 2).

#### 3.2.1. Discussion

This study validated a discharge disposition predictive tool using integer-based risk scores for patients at index hospitalization for stroke as well as its utility in reducing readmission rates. Of the patients who were discharged to home, the algorithm predicted 95.7% of them to have that discharge disposition. In the readmission analysis, the scenario of predicted discharge to home and actual discharge to home only had a readmission rate of 9.5%, which is well below the usual readmission rate for patients poststroke [16–18].

Creating predictive tools to better match patients with an appropriate discharge destination may decrease the transition time from admission to discharge, whether to home or facility. Clinicians may be able to better identify high-risk patients and initiate more complex discharge planning early in a patient's length of stay. Additionally, unnecessary readmissions may be prevented by matching a patient more accurately with their appropriate discharge location. Improved matching may result in fewer complications and better functional recovery. These predictive tools can be simple and quick to use and may decrease the length of stay and readmissions, thus reducing costs. The top five risk scores found to be predictive of discharge disposition were admission from an SNF, acute myocardial infarction, intracerebral hemorrhage, admission from “other” sources, and an age of 75 or older. Myocardial infarction and age of 75 or older are risk factors for stroke [22] and are common indicators for a more complex medical management [23]. Older patients are likely to have more comorbidities and less support at home compared to younger patients and may require further medical care and monitoring at a facility. Intracerebral hemorrhage expectedly has a high-risk score as it is considered more severe than ischemic stroke or transient ischemic attack as evidenced by its correlation with an increase in mortality [24].

We used readmission rates as an indicator of prediction tool quality due to the significance of this metric for hospital administrators and clinicians alike. Relevant literature encourages hospitals to take measures to identify high-risk patients for readmission and determine appropriate discharge disposition and follow-up services in order to reduce readmission rates [17, 18]. Readmission rates are a rising concern for both hospital administrators and clinicians alike. This common ground makes lowering unnecessary readmissions a high-priority focus among the healthcare team. Much research has explored predictors of readmission in conditions such as type 2 diabetes mellitus [12], stroke [17], heart failure, acute myocardial infarction, pneumonia, and chronic obstructive pulmonary disease [14]. Some of these admissions are often questioned as potentially preventable, and hospital staffs are encouraged to identify high-risk patients and intervene accordingly [16, 17]. This study contributed to the current literature by validating a predictive discharge disposition tool with readmission rates.

The predictive tool created in this study predicted home discharge with extremely high occurrence. This may have been due to the high scores attributed to admission source versus comorbidities. While it is clinically apparent that patients receiving medical management immediately prior to stroke are likely to require continued management after their short-term acute care hospital stay, this score may have diminished the effect of other variables that help distinguish the significance of factors such as comorbidities and lifestyle behaviors.

There were several limitations to this study. The findings are limited to two years of Medicare beneficiaries and may not be generalizable to all patients poststroke. Some patients may have lost insurance coverage after discharge, and their readmissions are not recorded in the CMS dataset. In future research, it would be beneficial to exclude those patients from the analysis cohort. Risk scores were calculated based on index hospitalization for stroke; however, we could not know if this was the patient's first stroke or if it was a recurrent stroke with the first stroke occurring prior to our dataset. Patients with recurrent strokes would likely be considered at higher risk for facility discharge; however, this could not be accounted for without a full admission history. When validating the predictive tool via readmission analysis, the threshold to determine when the algorithm would predict facility versus home discharge was arbitrarily set at 75% probability of facility discharge. However, this threshold could likely be adjusted to allow for a closer match between predicted and actual discharge dispositions. Collaborating with hospital administrators or physicians may allude to a more clinically meaningful threshold that increases confidence in relying on the predictive tool. The top risk score factor was the admission from an SNF, which was dramatically higher than the next highest factor. This score may have shifted the probability curve and resulted in high levels of predicted home discharge for patients admitted from any other source. Clinically, admission from an SNF indicates a patient with high medical management prestroke, and discharge back to a facility is assumed to be likely. Existing studies have pointed to decreased outcomes for patients admitted to SNFs in comparison to home [25] or IRF [3]. It is difficult to determine whether the disparity in outcomes is due to the patients' medical complexity or the type, quality, and amount of care received at an SNF. Because of this, admission source may not be an insightful variable that adds to the general clinical reasoning. Eliminating this variable may depress the risk scores and give greater weight toward comorbidities and stroke type. Additionally, details of each patient's characteristics are limited to the amount of detail in their claim. We did not track the role of factors such as functional status, treatments received during the acute care stay, or patient and family preference in determining discharge status. These factors may provide deeper insight into a patient's profile. Lastly, while readmission rates are a well-accepted measure of the quality of care, we are unable to distinguish if any given readmission was due to inappropriate discharge planning or poor quality of care along the patient's journey.

## 4. Conclusion

In this study, we developed a discharge disposition prediction tool for use after index hospitalization poststroke. We utilized a probabilistic model (logistic regression) to assess the relationship between the outcome variable (discharge status) and its predictors (patient characteristics). Regression coefficients were converted into risk scores to determine the probability of facility discharge using our probabilistic model. The advantage of using this model is the ability to generate both positive and negative scores. The discharge outcome was efficiently calculated by assigning risk scores to each patient. Many patients and hospital-related factors affect the discharge disposition, making it a complex decision-making process. Prediction tools are helpful to guide clinicians and hospital administrators as they seek ways to improve the quality of care and reduce preventable readmissions through efficient and appropriate discharge planning.

## Figures and Tables

**Table 1:** Demographic and clinical characteristics of patients with primary diagnosis of stroke by hospital discharge disposition status.

Patient characteristics	Home discharge (*N* = 31,625 (%))	Facility discharge (*N* = 42,800 (%))	*P* value
Sex			<0.0001
Male	16,038 (50.7)	18,728 (43.8)	
Female	15,587 (49.3)	24,072 (56.2)	
Age			<0.0001
18-64	3,636 (11.5)	3,439 (8.0)	
65-74	10,570 (33.4)	10,211 (23.9)	
≥75	17,419 (55.1)	29,150 (68.1)	
Race			<0.0001
White	24,952 (78.9)	33,604 (78.5)	
Black	4,565 (14.4)	6,710 (15.7)	
Other	2,108 (6.7)	2,486 (5.8)	
Stroke type			<0.0001
Ischemic	28,708 (90.8)	36,855 (86.1)	
Meningeal hemorrhage	1,215 (3.8)	1,698 (3.9)	
Intracerebral hemorrhage	1,702 (5.4)	4,247 (10.0)	
Comorbidity			<0.0001
Diabetes	10,300 (32.6)	14,569 (34.0)	
High cholesterol	17,300 (54.7)	22,333 (52.2)	
Obesity	3,078 (9.7)	4,018 (9.4)	
Hypertension	19,223 (60.8))	25,494 (59.6)	
Atrial fibrillation	9,134 (28.9)	15,551 (36.3)	
Other atrial disease	3,134 (9.9)	4,277 (10.0)	
Chronic kidney disease	5,372 (16.9	8,626 (20.2)	
Heart disease	12,599 (39.8)	19,171 (44.8)	
Peripheral arterial disease	1,904 (6.0)	2,749 (6.4)	
Other vascular	861 (2.7)	1,121 (2.6)	
TIA	9,104 (28.8)	13,239 (30.9)	
Acute heart attack	311 (1)	1,002 (2.3)	
Sleep habit	907 (2.9)	1,134 (2.6)	
Alcohol habit	936 (3.0)	1,354 (3.2)	
Drug habit	463 (1.5)	549 (1.3)	
Smoking	10,405 (32.9)	11,717 (27.4)	
Family history	2,373 (7.5)	2,351 (5.5)	
Depression	194 (0.6)	326 (0.8)	
Other diagnosis	819 (2.5)	1,572 (3.7)	
Source of admission			<0.0001
Nonhealthcare facility	28,641 (90.6)	36,911 (86.2)	
Clinic referral	1,172 (3.7)	1,443 (3.4)	
Transfer from a hospital	1,437 (4.5)	2,414 (5.6)	
Transfer from an SNF	138 (0.4)	1,463 (3.4)	
Other	237 (0.8)	569 (1.4)	
Type of insurance	13		
Medicare or Medicaid	30,585 (96.7)	42,129 (98.4)	
Private insurance	833 (2.6)	534 (1.2)	
Other	207 (0.7)	137 (0.4)	

**Table 2:** Total risk score calculation.

Beneficiary ID	Discharge status	Gender	Age	Stroke type	...	Total risk score
A	1	1	3	5	...	11
B	0	0	3	1	...	4
C	1	0	1	0	...	2

**Table 3:** Total risk score conversion.

Beneficiary ID	Total risk score	Actual discharge status (*y*)	(threshold = 9) Predicted discharge status ($\hat{y}$)
A	10	Facility	Facility
B	7	Facility	Home
C	14	Facility	Facility
D	4	Home	Home
E	9	Facility	Facility

**Table 4:** Odds ratios of patient characteristics associated with facility discharge.

Patient characteristics	Unadjusted OR	Adjusted OR	*β*
Sex			
Male	1.00 (Ref.)	1.00 (Ref.)	0
Female	1.32 (1.28-1.36)	1.25 (1.21-1.29)	0.2245
Age			
18-64	1.00 (Ref.)	1.00 (Ref.)	0
65-74	1.02 (0.97-1.08)	1.07 (1.01-1.13)	0.0692
≥75	1.77 (1.68-1.86)	1.71 (1.62-1.81)	0.5386
Race			
White	1.00 (Ref.)	1.00 (Ref.)	0
Black	1.09 (1.05-1.14)	1.21 (1.16-1.27)	0.1924
Other	0.88 (0.82-0.93)	0.91 (0.86-0.97)	-0.0942
Stroke type			
Ischemic	1.00 (Ref.)	1.00 (Ref.)	0
Meningeal hemorrhage	1.09 (1.01-1.17)	1.13 (1.05-1.22)	0.1239
Intracerebral hemorrhage	1.94 (1.83-2.06)	2.02 (1.90-2.14)	0.7020
Comorbidity			
Diabetes	1.07 (1.03-1.10)	1.15 (1.11-1.19)	0.1396
High cholesterol	0.89 (0.86-0.92)	0.92 (0.89-0.95)	-0.0806
Obesity	0.96 (0.92-1.01)	1.07 (1.01-1.12)	0.0647
Hypertension	1.08 (1.04-1.12)	1.05 (1.01-1.09)	0.0476
Atrial fibrillation	1.35 (1.31-1.39)	1.27 (1.23-1.31)	0.2386
Other atrial disease	1.01 (0.97-1.07)	1.06 (1.01-1.12)	0.0605
Chronic kidney disease	1.23 (1.17-1.29)	1.20 (1.14-1.26)	0.1791
Heart disease	1.13 (1.09-1.17)	1.11 (1.08-1.15)	0.1116
Peripheral arterial disease	1.04 (0.98-1.10)	1.04 (0.98-1.11)	0.0423
Other vascular	0.95 (0.87-1.04)	0.99 (0.90-1.09)	-0.0108
TIA	1.09 (1.06-1.13)	1.09 (1.05-1.12)	0.0822
Acute heart attack	2.22 (1.94-2.52)	2.24 (1.97-2.56)	0.8075
Sleep habit	0.95 (0.86-1.03)	0.97 (0.89-1.07)	-0.0277
Alcohol habit	1.21 (1.11-1.32)	1.44 (1.32-1.57)	0.3653
Drug habit	0.95 (0.84-1.08)	1.17 (1.02-1.33)	0.1585
Smoking	0.78 (0.76-0.81)	0.88 (0.85-0.91)	-0.1247
Family history	0.74 (0.70-0.79)	0.76 (0.72-0.81)	-0.2669
Depression	1.27 (1.01-1.52)	1.30 (1.08-1.57)	0.2640
Other diagnosis	1.43 (1.32-1.57)	1.42 (1.30-1.55)	0.3526
Source of admission			
Nonhealthcare facility	1.00 (Ref.)	1.00 (Ref.)	0
Clinic referral	0.95 (0.88-1.03)	0.97 (0.89-1.05)	-0.0342
Transfer from a hospital	1.30 (1.22-1.39)	1.24 (1.16-1.33)	0.2169
Transfer from an SNF	8.22 (6.90-9.80)	7.00 (5.87-8.35)	1.9461
Other	1.86 (1.60-2.17)	1.79 (1.53-2.09)	0.5820
Type of insurance			
Medicare or Medicaid	1.00 (Ref.)	1.00 (Ref.)	0
Private insurance	0.47 (0.42-0.52)	0.62 (0.55-0.69)	-0.4821
Other	0.48 (0.39-0.60)	0.69 (0.55-0.86)	-0.3703

**Table 5:** Risk scores of patient characteristics.

Patient characteristics	Risk score
Sex	
Male	0
Female	1
Age	
18-64	0
65-74	1
≥75	3
Race	
White	0
Black	1
Other	-1
Stroke type	
Ischemic	0
Meningeal hemorrhage	1
Intracerebral hemorrhage	5
Comorbidity	
Diabetes	1
High cholesterol	-1
Obesity	0
Hypertension	0
Atrial fibrillation	2
Other atrial diseases	0
Chronic kidney disease	1
Heart disease	1
Peripheral arterial disease	0
Other vascular	0
TIA	1
Acute heart attack	5
Sleep habit	0
Alcohol habit	2
Drug habit	1
Smoking	-1
Family history	-2
Depression	2
Other diagnosis	2
Source of admission	
Nonhealthcare facility	0
Clinic referral	0
Transfer from a hospital	1
Transfer from an SNF	13
Other	4
Type of insurance	
Medicare or Medicaid	0
Private insurance	-3
Other	-2

## Data Availability

The CMS data was purchased through ResDac. This data is not public since it contains patient privacy (HIPAA compliant).
